# Considerations and Consequences when using First Nations Identifiers in Administrative Data Research.

**DOI:** 10.23889/ijpds.v7i3.1939

**Published:** 2022-08-25

**Authors:** Anita Durksen, Wanda Phillips-Beck, Joykrishna Sarkar, Farzana Quddus, Jennifer Enns, Mariette Chartier, Nathan Nickel, Marni Brownell

**Affiliations:** 1 Manitoba Centre for Health Policy, University of Manitoba; 2 First Nations Health and Social Secretariat of Manitoba, University of Manitoba

## Objectives

Lack of consistent and relevant Indigenous identifiers in Canadian data sources leads to misclassification and under-recognition of the health and social issues impacting Indigenous Peoples, further perpetuating the harms of colonization. We are evaluating and optimizing our approach for identifying First Nations (FN) individuals in administrative data in Manitoba.

## Approach

In partnership between the First Nations Health and Social Secretariat of Manitoba and the Manitoba Centre for Health Policy, we sought to evaluate to what extent four distinct Manitoba datasets derived from surveillance and social programs were able to identify FN individuals among a cohort of children in low-income Winnipeg neighbourhoods between 2005-2016. In choosing data sources to identify FNs, the population and research question were considered.

We then compared the number of FNs identified in each dataset to the First Nations population research file, considered gold standard, but cognizant of its roots in colonial systems of registration.

## Results

The total cohort comprised N=78,864; among these, n=27,347 children were identified as FN in either the FN registry or at least one of the datasets. The FN registry was the most sensitive dataset and identified 85.7% of these individuals. The two program datasets identified 58.2% and 46.2%, and the surveillance-based datasets each identified less than 10%.

The First Nations Registry is critical to accurately identify FN individuals. Our analyses demonstrated that without it we would miss 23.5% of those identified as FN in at least one other dataset. However, using it alone could potentially cause us to miss 15% of FN individuals identified in other datasets.

The proportions of FN individuals that would be missed by excluding any of the other datasets were smaller (<6%).

## Conclusion

We found inconsistent FN identification across the datasets evaluated. Among the issues is that some datasets rely on self-identification. In Manitoba, no single administrative dataset can reliably and comprehensively identify FN individuals, and linking multiple datasets together is currently our best approach.

